# Genetic variation in DNA-repair pathways and response to radiochemotherapy in esophageal adenocarcinoma: a retrospective cohort study of the Eastern Cooperative Oncology Group

**DOI:** 10.1186/1471-2407-11-176

**Published:** 2011-05-17

**Authors:** Harry H Yoon, Paul J Catalano, Kathleen M Murphy, Todd C Skaar, Santosh Philips, Mark Powell, Elizabeth A Montgomery, Michael J Hafez, Steven M Offer, Geoffrey Liu, Stephen J Meltzer, Xifeng Wu, Arlene A Forastiere, Al B Benson, Lawrence R Kleinberg, Michael K Gibson

**Affiliations:** 1 Mayo Clinic, 200 First Street SW, Rochester, MN 55905, USA; 2 Dana-Farber Cancer Institute, 44 Binney Street, Boston, MA, 02115, USA; 3 Johns Hopkins University, 3400 North Charles Street, Baltimore, MD, 21218, USA; 4 Indiana University, 420 University Boulevard, Indianapolis, IN, 46202, USA; 5 Princess Margaret Hospital, 190 Elizabeth Street, Toronto, ON, M5G 2C4, Canada; 6 MD Anderson Cancer Center, The University of Texas, 1515 Holcombe Boulevard, Houston, TX, 77030, USA; 7 Northwestern University, 710 North Lake Shore Drive, Chicago, IL, 60611, USA; 8 University of Pittsburgh Medical Center, 5150 Centre Avenue, 5thfloor, Pittsburgh, PA, 15232, USA

## Abstract

**Background:**

Recent data in esophageal cancer suggests the variant allele of a single-nucleotide polymorphism (SNP) in *XRCC1 *may be associated with resistance to radiochemotherapy. However, this SNP has not been assessed in a histologically homogeneous clinical trial cohort that has been treated with a uniform approach. In addition, whether germline DNA may serve as a surrogate for tumor genotype at this locus is unknown in this disease. Our objective was to assess this SNP in relation to the pathologic complete response (pCR) rate in subjects with esophageal adenocarcinoma who received cisplatin-based preoperative radiochemotherapy in a multicenter clinical trial (Eastern Cooperative Oncology Group 1201). As a secondary aim, we investigated the rate of allelic imbalance between germline and tumor DNA.

**Methods:**

Eighty-one eligible treatment-naïve subjects with newly diagnosed resectable esophageal adenocarcinoma received radiotherapy (45 Gy) concurrent with cisplatin-based chemotherapy, with planned subsequent surgical resection. The primary endpoint was pCR, defined as complete absence of tumor in the surgical specimen after radiochemotherapy. Using germline DNA from 60 subjects, we examined the base-excision repair SNP, *XRCC1 *Arg399Gln, and 4 other SNPs in nucleotide excision (*XPD *Lys751Gln and Asp312Asn, *ERCC1 *3' flank) and double-stranded break (*XRCC2 *5' flank) repair pathways, and correlated genotype with pCR rate. Paired tumor tissue was used to estimate the frequency of allelic imbalance at the *XRCC1 *SNP.

**Results:**

The variant allele of the *XRCC1 *SNP (399Gln) was detected in 52% of subjects. Only 6% of subjects with the variant allele experienced a pCR, compared to 28% of subjects without the variant allele (odds ratio 5.37 for failing to achieve pCR, p = 0.062). Allelic imbalance at this locus was found in only 10% of informative subjects, suggesting that germline genotype may reflect tumor genotype at this locus. No significant association with pCR was noted for other SNPs.

**Conclusions:**

Assessed for the first time in a prospective, interventional trial cohort of esophageal adenocarcinoma, *XRCC1 *399Gln was associated with resistance to radiochemotherapy. Further investigation of this genetic variation is warranted in larger cohorts. In addition, these data indicate that germline genotype may serve as a surrogate for tumor genotype at this locus.

## Background

Esophageal adenocarcinoma (EAC) is one of the fastest rising cancers in the West, particularly among white men[1]. Survival remains poor despite experimentation with numerous cytotoxic agents and therapeutic approaches, as well as improvements in diagnostic, surgical, and radiation technique[2]. In the U.S., a widely used standard to attempt to cure locally advanced disease, which is a common stage at presentation, is to provide concurrent radiochemotherapy (RCT) followed by surgery. Cisplatin often forms the base of the chemotherapy regimen[3].

This approach causes considerable toxicity in the vast majority of patients. Moreover, therapies are given with little foreknowledge of outcome. Therefore, one method to improve outcomes in EAC is to identify which patients will respond best to a particular therapeutic approach or agent - or, conversely, to identify patients who will most likely fail standard therapy so as to deliver alternative therapy at or near the outset[4]. Such patient stratification holds the potential for maximizing efficacy and minimizing toxicity.

Current methods of patient stratification in the trimodality setting - *e.g.*, tumor grade, lymph node status, and other clinicopathologic traits - inadequately forecast clinical outcome. Therefore, the identification of biologic or molecular predictors is a rational step to tailor therapy according to an individual clinical-molecular profile.

Importantly, EAC represents a useful human model in which to study radiochemoresistance in gastrointestinal carcinomas. RCT leads to complete obliteration of tumor (*i.e.*, complete pathologic response [pCR]) in approximately 25% of EAC patients [5]. pCR has been repeatedly shown to be one of the strongest prognosticators of long-term outcome in EAC, associated with a 2- to 4-fold longer median overall survival[6-8]. Its importance is underscored by its increasing use as a primary endpoint in clinical trials for esophageal cancer. RCT typically leads to a higher pCR rate in EAC than in other gastrointestinal carcinomas, including those of the rectum[9] or pancreas[10].

Recent attention has focused on genetic variations in DNA repair pathways as a strategy for predicting response to DNA-damaging agents. DNA lesions induced by radiotherapy or cisplatin are repaired by the base excision (BER) and double-stranded break repair (DSBR) or by the nucleotide excision repair (NER) pathways, respectively. Key enzymes in these multistep complexes are XRCC1 (BER), XRCC2 (DSBR), and ERCC1 and XPD (both NER)[11]. It has been hypothesized that impaired BER/DSBR/NER activity in tumor cells leads to greater DNA damage after treatment with platins and/or ionizing radiation, thus causing greater tumor cell death[12-14]. Alternatively, greater DNA damage and genetic instability may produce tumor heterogeneity, giving rise to malignant clones that resist apoptosis after platin or radiation exposure[15].

Single nucleotide polymorphisms (SNP) in these pathways may alter DNA repair capacity. SNPs in the *XRCC1 *gene (Arg399Gln) and *XPD *gene (Lys751Gln) have been associated with increased DNA damage[16,17]. Studies of these SNPs in EAC are only recently emerging. Efforts to identify markers that predict radioresistance, which encompasses the radiosensitizing effects of chemotherapy, would focus naturally on BER or DSBR genes. A co-investigator (X.W.) found that the *XRCC1 *Arg399Gln SNP (BER) was associated with pCR in esophageal cancer patients treated with cisplatin-based RCT and surgery[18]. However, this cohort was retrospectively collected over a long period (1985-2003), received heterogeneous therapeutic approaches (*e.g.*, induction chemo prior to combined RCT were included), and combined squamous cell carcinoma (ESCC) and adenocarcinoma (EAC) histology. ESCC and EAC appear to be distinct from one another in epidemiology and biology[1,2,19]. Our objective in the current study was to build on this recent finding, by assessing this SNP and 4 others (*ERCC1, XPD, XRCC2*) in a cohort of only-EAC subjects, enrolled to a prospective, interventional clinical trial during a recent and short period and treated with cisplatin-based RCT in the first-line setting, without induction chemotherapy. We found that the relationship between the *XRCC1 *SNP and pCR pointed in the same direction as found by our co-investigator. To our knowledge, this is the first assessment of this SNP in EAC patients receiving RCT in a prospective clinical trial.

In addition, we assessed whether germline genotype may serve as a surrogate for tumor genotype at this *XRCC1 *SNP by examining allelic imbalance (AI). AI is the loss or gain of a DNA region in tumor (as compared to germline) cells. This issue has relevance in both the research and clinical context. Studies evaluating the relation between SNPs and therapeutic efficacy in humans have typically studied germline DNA (peripheral blood lymphocytes or normal tissue) under the assumption that germline genotype usually reflects tumor genotype (*i.e.*, that AI is rare at these loci). However, the rate of AI in resectable EAC at this *XRCC1 *SNP is unknown. A low rate of AI would provide biologic plausibility that germline genotype may be used as a surrogate for tumor genotype at these loci in predicting treatment response. Therefore, we examined tumor tissues with matched histologically normal tissues to assess AI. We found that AI at this *XRCC1 *SNP is uncommon; this finding supports the use of germline DNA, which is far more accessible clinically, when attempting to use this SNP to predict therapeutic tumor response in the tumor using this SNP to therapy.

## Methods

### Subjects, endpoints

DNA was obtained from subjects enrolled in ECOG trial E1201. Briefly, E1201 was a multicenter, randomized phase II trial (2002-04) that enrolled treatment-naïve subjects with newly diagnosed adenocarcinoma of esophagus or gastroesophageal junction (tumor extension ≤2 cm into gastric cardia)[20]. Other eligibility criteria included: locally advanced stage (*i.e.*, T_2-3_N_0_M_0_, T_1-3 _N_1_M_0 _or T_1-3_N_0-1_M_1a_), surgically resectable disease (T_1-3 _but not T_4_), ECOG performance status 0-1, and staging by endoscopic ultrasound with esophagogastroduodenoscopy and CT of the chest and abdomen. Subjects in both arms received radiotherapy to 45 Gy administered at 1.8 Gy per day, 5 days per week for 5 weeks, concurrently with chemotherapy. One arm received cisplatin 30 mg/m^2 ^days 1, 8, 22, 29, and irinotecan 65 mg/m^2 ^days 1, 8, 22, 29. The other arm received cisplatin 30 mg/m^2 ^days 1, 8, 15, 22, 29, and paclitaxel 50 mg/m^2 ^(1 hr) days 1, 8, 15, 22, 29. Subjects in both arms underwent surgical resection approximately 5 weeks after the completion of RCT. The primary endpoint of E1201 as well as the current study was pCR - *i.e.*, the complete absence of tumor in the resected specimen subsequent to RCT; pCR was assessed by local geographic sites during E1201, not by central review. Secondary endpoints were progression-free survival (PFS) and overall survival (OS).

### Retrieval and processing of specimens

Stored paraffin-embedded tissue specimens were obtained from the ECOG Pathology Coordinating Office. Both pretreatment biopsies and posttreatment resection samples were obtained for each subject whenever available. Fresh hematoxylin and eosin-stained sections (H&Es) were generated, then marked by an esophageal anatomic pathologist (E.A.M.) for areas containing only histologically non-malignant tissue or areas enriched (>60%) in tumor cells. Areas of Barrett's metaplasia and dysplasia were avoided. These H&Es were used as references for macrodissection of unstained slides. DNA was extracted from macrodissected specimens using the Qiagen QIAamp DNA FFPE Tissue Kit (Valencia, CA) following manufacturer's instructions

### Genotyping

The Arg399Gln SNP within the X-ray repair complementing defective repair in Chinese hamster cells 1 (*XRCC1*) gene was chosen based on its prior association with complete pathologic response in esophageal cancer patients treated with RCT[18]. Other SNPs were selected if: (1) residing in a NER, BER, or DSBR pathway gene; and (2) previously associated with clinical outcome in solid-tumor patients treated with platinum-based chemotherapy[12,21-23] or with cancer risk[24]. Four additional SNPs were identified in 3 genes: excision repair cross-complementing rodent repair deficiency, complementation group 2 (*XPD/ERCC2*, Lys751Gln and Asp312Asn); excision repair cross-complementing rodent repair deficiency, complementation group 1 (*ERCC1 *3' flank, also known as "C8092A"); X-ray repair complementing defective repair in Chinese hamster cells 2 (*XRCC2 *5' flank, also known as "-7985T > C"). PCR and extension primers were designed on the Sequenom Assay Designer 3.1 Software (San Diego, CA) based on sequences available through the National Center for Biotechnology Information (dbSNP; http://www.ncbi.nlm.nih.gov/projects/SNP)). Allelotyping was performed using mass spectrometry-based allelotyping software and matrix-assisted laser desorption ionization-time of flight spectrometry (MALDITOF; MassArray System, Sequenom, San Diego, CA) following the manufacturer's instructions[25]. All samples were run in triplicate. The analysis of the spectra was done using the Sequenom Allelotyping Software Typer Version 4.0 (San Diego, CA; http://www.sequenom.com). MALDITOF was used to determine allelic imbalance at the *XRCC1 *Arg399Gln loci by dividing the allele frequency ratio of the tumor sample by the allele frequency ratio of the corresponding normal sample[26,27]. A sample was scored as showing allelic imbalance if this quotient was ≤0.6 or ≥1.67 (indicating that one of the alleles had decreased 40% or more)[26,27].

### Analytic and statistical approach

Data was pooled across both arms of the parent study and analyzed at ECOG. Lab investigators remained blinded to individual subject data throughout the study, including during data analysis. Genotypes were dichotomized *a priori *into 2 groups: (1) major homozygote or (2) variant allele group (heterozygote or variant-allele homozygote)[18]. Univariate comparisons were made between each SNP and clinical endpoint. Multivariate adjustments were not performed because covariates (*e.g.*, age, performance status) were not associated with any endpoint in univariate analyses. Exact logistic regression was used to estimate odds ratios (OR) and 95% confidence intervals (CI). All p-values and CIs are two-sided. Analyses were done in SAS version 8.2 (Cary, NC) for UNIX (SunOS). Using R^2 ^correlation (Haploview software 4.2, broadinstitute.org), linkage was assessed among the four SNPs on 19q (*XPD, ERCC1, XRCC1*). R^2 ^≥0.80 were considered significant. Hardy Weinberg equilibrium analysis for each SNP was calculated using R statistical software (http://www.r-project.org).

The study was approved by the ECOG Laboratory Science Committee, ECOG Gastrointestinal Committee, and the Institutional Review Boards of Johns Hopkins and Mayo Clinic. All subjects provided informed consent.

## Results

### Assembly and description of cohort

**Figure 1:**
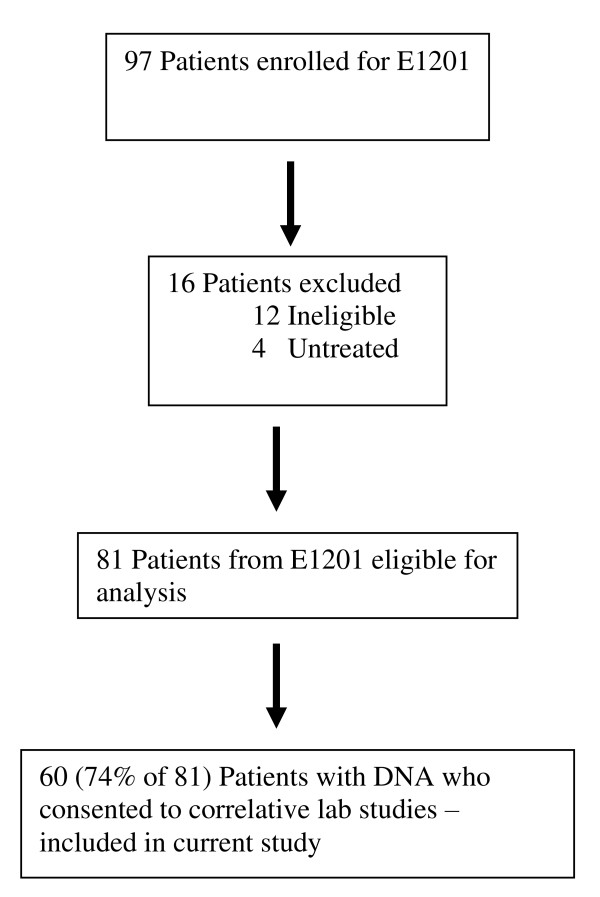
**Patient eligibility in E1201 and sample availability**.

Of 81 eligible subjects in E1201, 60 (74%) consented to correlative lab studies and had sufficient DNA. These 60 subjects form the primary study population of the current analysis (Figure 1). Baseline clinicopathologic traits were: median age 57 years (38-76); male 88%; white 93%, black 2%, Hispanic 2%, Asian 3%; node-negative (T_2-3_N_0_M_0_) 28%, node-positive (T_1-3 _N_1_M_0 _or T_1-3_N_0-1_M_1a_) 72%; ECOG performance status 0 (65%) and 1 (35%); mid- (2%) and lower thoracic (45%) esophagus, gastroesophageal junction (48%), esophagus not otherwise specified (3%), and unknown (2%). Ten subjects achieved a pCR. The clinicopathologic features of the current study population are similar to those of the parent E1201 cohort (data not shown). Median PFS and OS were 46.5 and 46.7 months, respectively.

### Germline genotype and outcome

All subjects were successfully genotyped for all evaluated SNPs. The variant allele of the *XRCC1 *SNP (399Gln) was detected in 52% of subjects. As shown in Table 1, only 6% of subjects with the variant allele experienced a pCR, compared to 28% of subjects without the variant allele (OR 5.37 for failing to achieve pCR [95% CI 0.94, 57.0], p = 0.062). No significant association with pCR was noted for other SNPs, and no SNP was associated with PFS or OS (Table 2). Paired tumor tissue was available in 21 of 22 informative (germline heterozygote) subjects. AI was present in 2 (10%) of 21 informative subjects. No significant linkage was noted for the SNPs genotyped on 19q.

**Table 1:** Genotype and therapeutic response

	Baseline Frequency		Association between SNPs and pCR				
		
SNP	Geno-type	No. (%)	**Variant allele group**^**a **^***vs *major homozygote**	No pCR/pCR (No.)	OR	95% CI	*p*
*XRCC1 *Arg399Gln (rs25487)	AA	12 (20)					
	AG	19 (32)	AA or AG	29/2			
	GG	29 (48)	GG	21/8	5.37	0.94-57.0	0.062

*XRCC2 *5' flank (rs6464268)	GG	1 (2)					
	GA	16 (27)	GG or GA	13/4			
	AA	43 (72)	AA	37/6	0.53	0.11-2.99	0.59

*ERCC1 *3' flank (rs3212986)	TT	0					
	GT	22 (37)	GT	17/5			
	GG	38 (63)	GG	33/5	0.52	0.10-2.60	0.54

*XPD *Lys751Gln (rs13181)	GG	8 (13)					
	GT	29 (48)	GG or GT	30/7			
	TT	23 (38)	TT	20/3	0.64	0.10-3.3	0.83

*XPD *Asp312Asn (rs1799793)	AA	7 (12)					
	AG	28 (47)	AA or AG	28/7			
	GG	25 (42)	GG	22/3	0.55	0.082-2.77	0.65

**CI**, confidence interval; **OR**, odds ratio for non-pCR of variant allele group as compared to the major homozygote; **pCR**, complete pathologic response; **SNP**, single nucleotide polymorphism.

^a ^The variant allele group consists of the variant allele homozygote and heterozygote in combination.

**Table 2:** Genotype and survival

	Progression-free survival			Overall Survival		
		
SNP and Genotype	Median (months)	HR	*p*	Median (months)	HR	*p*
*XRCC1*:rs25487						
AA or AG	31.5	1.4	0.41	23.7	0.86	0.66
GG	NR (>38)			49.3		

*XRCC2*:rs6464628						
GG or GA	22.9	1.6	0.24	53.5	1.3	0.52
AA	NR (>31.5)			46.7		

*ERCC1*:rs3212986						
GT or TT	37.9	1.1	0.78	46.7	0.96	0.90
GG	NR (>31.5)			35.0		

*XPD*:rs13181						
GG or GT	46.5	0.63	0.25	46.7	0.67	0.27
TT	16.0			21.0		

*XPD*:rs1799793						
AA or AG	NR (>40)	0.80	0.57	NR (>40)	0.59	0.14
GG	31.4			21.0		

**HR**, hazard ratio; **NR**, not reached; **SNP**, single nucleotide polymorphism.

Hardy-Weinberg analysis was performed in 68 subjects total, consisting of the 60 patients from the current study cohort and 8 additional subjects who enrolled in E1201 but were later deemed ineligible for the parent study. All SNPs were in Hardy-Weinberg equilibrium (p > 0.15) except *XRCC1 *Arg399Gln (p = 0.028). For *XRCC1*, the expected *vs *observed genotype frequencies (*i.e.*, number of subjects) were 28.5 *vs *33 for GG, 31.1 *vs *22 for AG, and 8.5 *vs *13 for AA.

## Discussion

The ability to predict, in the pre-RCT setting, which human EACs are radiochemosensitive has enormous clinical relevance. This is because RCT carries substantial toxicity, and because preoperative chemotherapy alone, without radiotherapy, is a valid curative alternative to RCT[28,29]. The likelihood that a patient's tumor is radiochemosensitive may be incorporated into a risk/benefit model at the time of diagnosis which could be used as a clinical decision-making tool to select patients for preoperative RCT *vs *chemotherapy alone. pCR is a clinically relevant marker of radiochemosensitivity, due to its consistent and strong association with overall survival in this disease[4]. Because traditional clinical-pathologic factors in the pretreatment setting do not adequately predict radiochemosensitivity, it is reasonable to evaluate molecular or genetic factors[4,19].

In this study we found that 52% of EAC subjects had the variant 399Gln allele in *XRCC1*, and that subjects with the variant allele had five times higher odds of failing to achieve pCR after cisplatin-based RCT, compared to subjects without the variant allele. To our knowledge, our study is the first to assess this SNP in relation to radiochemoresistance in a clinical trial cohort of EAC. This association did not reach statistical significance at the 0.05 alpha level, which may reflect our relatively modest sample size. A number of observations support the probability that our observed findings are real, and would have reached greater statistical significance had more samples been available for analysis. One, the only other study we are aware of which assessed the relationship between this SNP in a cohort of mostly EAC patients receiving platin-based RCT and pCR (co-investigator X.W.) found the same association. Our cohort was completely independent from the first and was analyzed using a different genotyping platform.

Two, our study employed strong methodology. Therapeutic approach, staging, and disease characteristics were highly uniform, well-characterized, and prospectively collected. Our subjects were accrued over a recent, short time period across multiple centers, and staged by modern methods. Only adenocarcinoma histology was included. We focused on adenocarcinoma because of its greater relevance in the U.S., and the epidemiologic and potential biologic differences compared to squamous cell carcinoma[19]. Lab investigators remained blinded to clinical outcomes.

Three, we found that tumor genotype reflected germline genotype at the *XRCC1 *399 loci (19q13.2) in 90% of informative cases, suggesting germline DNA may be an appropriate surrogate of tumor genotype. To our knowledge, this is the first reported assessment of AI specifically in *XRCC1 *in EAC. Comparative genomic hybridization (CGH) studies of EAC have not found substantial genetic alterations in this general region[30,31]. One study found >10% of 28 GEJ cancers (including 3 cell lines and 2 xenografts) had gene amplification at a neighboring site (19q13.1) [31]; however, the density of CGH coverage is not clearly reported [32] so it is unknown whether amplification occurred at *XRCC1*. Together, these AI data, which require further evaluation and confirmation, provides biologic plausibility that assessing germline DNA is appropriate when attempting to predict response to RCT in EAC tumors.

In addition, *XRCC1 *is a key player in BER, the major repair pathway for nonbulky damaged bases, abasic sites, and DNA single-stranded breaks after treatment with ionizing radiation[33,34]. Prior reports in human populations suggested the 399Gln variant of *XRCC1 *was associated with greater DNA and chromosomal damage[16,35]. Worsened pCR and survival[18] related to the variant may be due to increased genetic instability and the development of multiple clonal populations, given the substantial data linking chromosomal aberrations and poor prognosis[15,18]. One co-investigator (G.L.) assessed this *XRCC1 *SNP in patients with esophageal cancer treated with cisplatin-based trimodality therapy and, similar to the current study, did not find an association with disease-free or overall survival[36]. Also consistent with our findings, the 399Gln variant allele was associated with decreased tumor response (and worse survival) in patients with stage III-IV non-small cell lung cancer (NSCLC) and metastatic colorectal cancer, respectively, treated with platinum chemotherapy[22,37]. By contrast, the variant allele was associated with favorable OS in patients with stage IV squamous cell carcinoma of the head and neck (SCCHN) treated with cisplatin-based chemotherapy or RCT[12]. Investigators of the latter study hypothesized their divergent result may have been due to biologic or tissue-specific factors underlying the greater chemosensitivity of SCCHN, compared to lung cancer, or to the differing types of platin compounds used in other studies (mostly carboplatin in NSCLC and oxaliplatin in colorectal cancer).

The *XRCC1 *SNP deviated somewhat from Hardy-Weinberg equilibrium, with under-representation of the heterozygote genotype *vs *over-representation of one of the homozygous genotypes. This raises the possibility that one of the homozygous genotypes increases EAC risk, as supported by some [38] but not other [39,40] case-control data; however, our study design does not enable firm conclusions to be drawn.

Because this study focused on previously reported alleles in a few genes, we have not accounted for the potential influence of other SNPs on clinical outcome. In addition, because the two arms of the study were pooled, the study does not account for the potential differential effects of the second chemoagent of each arm. While the effect size noted in our study between the *XRCC1 *SNP and pCR was considerable, the results did not reach statistical significance at the alpha 0.05 level; therefore, our results should be viewed with caution pending validation in an independent cohort. Despite substantial effort to identify prognostic or predictive biomarkers in EAC, the process remains in its early stages. Because chemo- or radiotherapy deliver their effects through multigenic steps, it is unlikely that a single SNP or marker will robustly predict therapeutic efficacy. In the practical clinical context, a combination of markers will likely be required. Our finding supports the further evaluation of this *XRCC1 *SNP in larger cohorts.

## Conclusion

EAC subjects with the variant 399Gln allele in *XRCC1 *had higher odds of radiochemoresistance compared to subjects without the variant allele (p = 0.062). To our knowledge, our study is the first to assess this SNP in relation to radiochemoresistance in a clinical trial cohort of EAC. These data support further investigation of this SNP in larger EAC populations. In addition, our data indicate that germline genotype may serve as a surrogate for tumor genotype at this locus.

## Competing interests

The authors declare that they have no competing interests.

## Authors' contributions

The following authors were involved in study design: HHY, MKG, ABB, AAF, KMM, GL, XW, TCK, SJM, LRK, MP, and PJC. HHY, KMM, MJH, SMO, AAF, XW, GL, SJM, EAC, SP, and TCK contributed to execution and interpretation of lab assays and/or interpretation of study results. Statistical analysis was performed by PJC and MP. All authors critically reviewed and approved the final manuscript.

## Author Information for Some Authors

• HHY, lead investigator of current study, Assistant Professor of Oncology, Mayo Clinic

• MKG, lead co-investigator of current study, Assistant Professor of Medicine, Univ. of Pittsburgh

• LRK, principal investigator of E1201 parent study, Associate Professor of Radiation Oncology and Molecular Radiation Sciences, Associate Professor of Oncology, Associate Professor of Neurological Surgery, Johns Hopkins Medical Center.

• ABB, chair of ECOG-GI committee, Professor in Medicine-Hematology/Oncology, Northwestern University Medical School

• PJC and MP, ECOG biostatisticians

• XW, lead author of prior XRCC1 SNP study, Professor, Department of Epidemiology, Division of Cancer Prevention and Population Sciences, The University of Texas M. D. Anderson Cancer Center

## Pre-publication history

The pre-publication history for this paper can be accessed here:

http://www.biomedcentral.com/1471-2407/11/176/prepub
